# Measuring research mistrust in adolescents and adults: Validity and reliability of an adapted version of the Group-Based Medical Mistrust Scale

**DOI:** 10.1371/journal.pone.0245783

**Published:** 2021-01-22

**Authors:** Amelia S. Knopf, Peter Krombach, Amy J. Katz, Rebecca Baker, Gregory Zimet

**Affiliations:** 1 Department of Community and Health Systems, Indiana University School of Nursing, Indianapolis, Indiana, United States of America; 2 Office of Evaluation, Indiana University School of Nursing, Indianapolis, Indiana, United States of America; 3 Department of Pediatrics, Division of Adolescent Medicine, Indiana University School of Medicine, Indianapolis, Indiana, United States of America; George Washington University, UNITED STATES

## Abstract

Mistrust of health care providers among persons of color is a significant barrier to engaging them in research studies. Underrepresentation of persons of color is particularly problematic when the health problem under study disproportionately affects minoritized communities. The purpose of this study was to test the validity and reliability of an abbreviated and adapted version of the Group Based Medical Mistrust Scale. The GBMMS is a 12-item scale with three subscales that assess suspicion, experiences of discrimination, and lack of support in the health care setting. To adapt for use in the research setting, we shortened the scale to six items, and replaced “health care workers” and “health care” with “medical researchers” and “medical research,” respectively. Using panelists from a market research firm, we recruited and enrolled a racially and ethnically diverse sample of American adults (N = 365) and adolescents aged 14–17 (N = 250). We administered the adapted scale in a web-based survey. We used Cronbach’s alpha to evaluate measure internal reliability of the scale and external factor analysis to evaluate the relationships between the revised scale items. Five of the six items loaded onto a single factor, with (α = 0.917) for adolescents and (α = 0.912) for adults. Mean scores for each item ranged from 2.5–2.9, and the mean summary score (range 6–25) was 13.3 for adults and 13.1 for adolescents. Among adults, Black respondents had significantly higher mean summary scores compared to whites and those in other racia/ethnic groups (p<0.001). There was a trend toward significance for Black adolescents as compared to white respondents and those in other racial/ethnic groups (p = 0.09). This five-item modified version of the GBMMS is reliable and valid for measuring research mistrust with American adults and adolescents of diverse racial and ethnic identities.

## Introduction

Mistrust of medical research and researchers create challenges for recruiting and enrolling participants in biomedical research studies. Mistrust of health care providers and health researchers are relevant to multiple racial and ethnic minority groups, but are particularly salient for Black Americans [1], due to a long history of being subject to unethical and abusive research [2, 3] as well as racially-biased treatment in health care settings [4]. Historical and ongoing mistreatment has led to underrepresentation of Black Americans in clinical research despite federal guidelines to ensure their engagement in federally funded research [5, 6].

Studies of the role of mistrust of health care providers and researchers in Black Americans’ and other racial and ethnic minority persons’ willingness to participate in clinical trials have had mixed results. Some research has indicated that mistrust is the most significant barrier to participation in clinical research [5, 7, 8]. George et al. [7] conducted a systematic review of barriers and facilitators of research participation among racial and ethnic minorities and found mistrust was the most common barrier to participation. Similarly, Luebbert et al. [9] reviewed 20 studies on barriers to research participation, and reported mistrust and concerns about experimentation as two of four major barriers identified by Black Americans. Other studies have indicated a broader range and prioritization of barriers to research participation. Newman et al. [10] reported fear of vaccine-induced HIV infection and concerns about side effects and vaccine efficacy were more frequently reported barriers to participation in HIV vaccine trials than was mistrust. Other commonly identified barriers have included fear of needles, concerns about long-term consequences of biomedical research participation, and logistical aspects of taking part in clinical studies [11].

National Institutes of Health funding guidelines not only emphasize the importance of enrolling a racially and ethnically diverse group of participants, but also the need to include children and adolescents, when scientifically appropriate [12]. However, the relationship between mistrust and willingness to participate in medical research is not well explored among adolescents who might participate in clinical trials or their parents. Understanding this relationship is particularly critical in the context of biomedical research on health problems that disproportionately affect adolescents and youth of color. For example, Black adolescents and youth bear a disproportionate burden of HIV incidence in the U.S. [13]. Recent developments in biomedical HIV prevention, including the use of oral and injectable anti-retrovirals as pre-exposure prophylaxis (PrEP) for HIV, have prompted a national and global interest in engaging adolescents in biomedical trials for HIV prevention [14–17]. However, recruiting and enrolling adolescents in clinical trials for sensitive and stigmatizing health problems is profoundly difficult [18, 19]. The underrepresentation of adolescents–particularly adolescents of color—in biomedical trials has important implications for resolving disparities in the health problems that disproportionately affect them. Our team received federal funding to conduct a study (*Consent 2*.*0*, Adolescent Medicine Trials Network for HIV/AIDS Interventions protocol 150) of barriers to minor adolescents’ research participation in HIV trials [20]. Medical mistrust was a covariate of interest; we wanted to describe the prevalence and severity of research mistrust among adolescents and parents of adolescents, and the relationship between mistrust and participation in HIV trials.

In preparation for an HIV research trial, our team reviewed scales that measure medical and research mistrust [21–24]. We determined the Group-Based Medical Mistrust Scale [24] (GBMMS; described in detail below) was likely to be the most accessible our future trial participants whose age range was quite broad (ages 13 and older). The GBMMS items are short and clear, making it an ideal scale to use. However, the scale is largely focused on medical care, not research, and it refers only to respondents’ ethnic group, and not racial identity. Therefore, we elected to adapt the GBMMS for use with our target population. In this paper we report on the psychometric properties of the adapted GBMMS.

## Materials and methods

### Study sample and procedures

The study was approved by the institutional review board at Indiana University and granted exempt status on 3/29/2017 (Protocol number 1703867613). Eligibility criteria were designed to match our target population for a future trial [20]. Participants were either: a) an adult who is parenting a 14–17 year-old and at least 29 years-old (age for parenthood of a teen) or b) an adolescent aged 14–17 years, inclusive; adult participants and adolescent participants were not related. All participants were living in the United States at the time of the research. The screening survey for both adults and minor adolescents began with a study information sheet that provided all elements of informed consent, including study purpose, study procedures, risks, benefits, confidentiality, voluntariness, and contact information for questions and concerns. At the end of the study information sheet, participants were informed to click forward to advance to the screener, which indicated consent to participate. Minor adolescents were recruited directly by parents, whose consent was implied by this recruitment. Data were collected in April 2017. Participants were recruited by Survey Sampling International (SSI; now Dynata), a market research firm that maintains panels of 62 million volunteer survey respondents throughout 100 countries. Panelists receive monetary incentives tailored to both the time and effort required for participation and regional preferences. SSI does not maintain active adolescent panels, so it distributed email invitations to parents of adolescents and asked them to send the survey link to their adolescent if participation was acceptable to both the parent and the adolescent.

### Measures

#### Demographics

All participants were asked to indicate their gender (female, male, transgender); their race (American Indian, Asian, Black, Native Hawaiian or Alaska Native, White, Other; choose all that apply); their ethnicity (Hispanic or non-Hispanic), and household size. Measures of socioeconomic status included: annual household income (adults only), the Family Affluence Scale (FAS-III; adolescents only), and current level of education (adolescents only).

#### The Group-Based Medical Mistrust Scale

The GBMMS was developed to measure medical mistrust and its relationship between breast cancer screening and treatment among Black and Latina women [24]. The scale has 12 5-point Likert-type items that load onto three factors, creating three subscales: suspicion of doctors and health care workers, disparities in health care, and lack of support from health care providers. The suspicion subscale includes questions such as *“People of my ethnic group should be suspicious of information from doctors and health care workers*.*”* The Disparities in health care subscale includes questions like *“People of my ethnic group receive the same medical care from doctors and health care workers as people from other groups*.*”* Lack of support from health care providers is assessed by questions like *“Doctors have the best interests of people of my ethnic group in mind*.*”*

The original GBMMS assumes contact with the health care system. We planned to use the scale to measure mistrust in health researchers and health research more generally. Contact and experiences with health care providers is common, whereas contact with researchers and experiences in research settings are not which would make it difficult for the majority of respondents to answer questions about experiences of disparity and lack of support. Thus, items that assumed prior experience with researchers/research were deemed inapplicable. For example, we removed items like “*In most hospitals*, *people of different ethnic groups receive the same kind of care*,” and “*I have personally been treated poorly or unfairly by doctors or health care workers because of my ethnicity*.” Ultimately, we selected six questions which were the most relevant to our broader research goals, and applicable to respondents who may not have participated in research before. Next, we replaced medicine or health care with research-related terms. Finally, we expanded beyond ethnic group to include racial identity as well. Adaptations are reflected in Table 1. With the minor revisions, the final version of the GBMMS consisted of 6 items with response choices ranging from 1 (Strongly Disagree) to 5 (Strongly Agree).

**Table 1:** Original and adapted items.

Original Item	Adapted Item
People of my ethnic group cannot trust doctors and health care workers.	People of my racial/ethnic group should not trust medical researchers.
People of my ethnic group should be suspicious of information from doctors and health care workers.	People of my racial/ethnic group should be suspicious of information from medical researchers.
People of my ethnic group should be suspicious of modern medicine.	People of my racial/ethnic group should be suspicious of medical research.
Doctors and health care workers treat people of my ethnic group like “guinea pigs.”	Medical researchers treat people of my racial/ethnic group like guinea pigs.
Doctors and health care workers sometimes hide information from people who belong to my ethnic group.	Medical researchers hide information from my racial/ethnic group.
People of my ethnic group are treated the same as people of other groups by doctors and health care workers.*	Medical researchers treat people of my racial/ethnic group the same as other racial/ethnic groups.*

*Reverse scored

### Data collection

The adapted scale was programmed for computer-assisted self-interview (CASI) using REDCap, a secure web application for building and managing online surveys. Panelists received an email invitation to participate in the survey. Once they clicked on the “start survey” button, they were taken to a study information page that described the study purpose, risks, benefits, and its voluntary nature. Panelists were instructed to click “next” if they were interested in screening for eligibility. Panelists were immediately notified of eligibility status, and, if eligible, were instructed to click “next” if they wished to complete the survey. Data were stored in REDCap and then imported into SPSS for analysis (version 26).

### Analysis

Descriptive statistics, including demographics and population characteristics, are reported in Table 2 as percentages for categorical variables and means and standard deviations for continuous variables. The mean and standard deviation of each individual item is represented in Table 3, stratified by participant group. Cronbach’s α was used to measure internal reliability for both adolescents and adults, and exploratory factor analysis (EFA) was used to evaluate the relationships between items in the revised scale and determine whether the items reflected one or more underlying constructs. Principal component analysis with Equimax rotation was used to yield factor loadings for each rotated component as seen in the pattern matrices outlined in Table 3. Confirmatory factor analysis was not conducted given our assumptions that we had substantially modified the original GBMMS scale from 12 items to 6 items, and that this modified scale may not fit the original 3-subscale structure.

**Table 2:** Demographic characteristics.

	Adults (N = 365)		Adolescents (N = 250)	
**Age**				
	Mean = 41	SD = 7.5	14	13%
			15	27%
			16	30%
			17	30%
	**N**	**%**	**N**	**%**
**Gender**				
Female	251	69%	136	55%
Male	111	30%	113	45%
transgender	2	1%	0	-
**Race**				
American Indian	12	3%	6	2%
Asian	6	2%	8	3%
Black	117	32%	56	23%
Native Hawaiian or Alaska Native	2	<1%	0	-
White	208	57%	174	70%
Other Race	20	6%	6	2%
**Hispanic**				
Yes	124	34%	69	28%
**Household size**	Mean = 4	SD = 1.3	N/A	N/A
**Household Income**				
Less than $14,999	22	6%	N/A	N/A
$15,000–29,999	42	12%	N/A	N/A
$30,000–49,999	79	22%	N/A	N/A
$50,000–74,999	85	23%	N/A	N/A
$75,000 or more	137	37%	N/A	N/A

**Table 3:** EFA standardize factor loadings for 6-item scale for adults and adolescents.

	Mean (SD)	Factor Loadings	Mean (SD)	Factor Loadings	
		Component 1		Component 1	Component 2
People of my racial/ethnic group should not trust medical researchers.	2.5 (1.1)	0.848	2.5 (1.2)	0.857	0.003
People of my racial/ethnic group should be suspicious of information from medical researchers.	2.7 (1.1)	0.87	2.6 (1.2)	0.903	-0.016
People of my racial/ethnic group should be suspicious of medical research.	2.6 (1.1)	0.881	2.6 (1.2)	0.879	0.097
Medical researchers treat people of my racial/ethnic group like guinea pigs.	2.7 (1.2)	0.872	2.6 (1.1)	0.863	0.032
Medical researchers hide information from my racial/ethnic group.	2.9 (1.2)	0.823	2.8 (1.2)	0.830	-0.012
Medical researchers treat people of my racial/ethnic group the same as other ethnic groups.	3.4 (1.2)	0.208	3.5 (1.1)	0.022	0.998

A cumulative medical mistrust score (with higher scores indicative of more mistrust) was created for both adolescents and adults, as seen in Table 4. Bivariate analysis (ANOVA, t-tests, independent t-tests) assessed differences in the total medical mistrust score by population type (adolescent vs adult), gender (male vs female) and racial/ethnic groups (Black vs white, non-Hispanic vs other) with statistical significance set at p-values <0.05. Although the p-value for differences between racial/ethnic groups met our significance threshold for adults, we did not perform post-hoc assessments for differences between these specific race groups given the heterogeneity of specific race groups.

**Table 4:** Research mistrust summary scores: Adults and adolescents, by race & ethnicity.

	Adults			Adolescent		
Characteristic	Mean	Std Dev	P-value	Mean	Std Dev	P-value
*Race/Ethnicity*			< .001			0.089
Black	15.1	4.4		14.4	3.2	
White, Non-Hispanic	11.5	4.9		12.8	5.6	
Other	13.4	4.9		12.7	4.9	
*Gender Identity*			0.114			0.729
Male	14.0	5.2		13.3	5.0	
Female	13.0	4.9		13.0	5.1	

## Results

### Response rate

#### Adults

A total of 544 SSI panelists responded to the parent survey; of these, 414 (76%) completed the survey. Respondents who reported their age was less than 29 or clearly mistyped their age (e.g. 1965) were dropped from the sample, resulting in a final sample of 365.

#### Adolescents

A total of 1208 SSI panelists began the adolescent survey; of these, 865 completed the survey, and 262 were eligible to participate. Age (younger than 14 or older than 17) was most common reason for ineligibility. After removing duplicates, the final sample size was 250. Demographic characteristics are described in Table 2.

### Research mistrust

#### Factor analysis

For adults and adolescents, five of the six items loaded onto a single factor. For adolescents, the reverse-coded item, ‘*Medical researchers treat people of my racial/ethnic group the same as other racial/ethnic groups*’, loaded onto its own factor (Table 3) for adolescents, but not for adults. Loadings ranged from 0.83 to 0.90 and 0.82 to 0.88 for adolescents and adults, respectively. With all six items included, the scale was still reliable for both adolescents (α = 0.848) and adults (α = 0.853). However, the reliability improved substantially with this item removed for both adolescents (α = 0.917) and adults (α = 0.912).

#### Descriptive statistics

Mean scores for each item in the revised 5-item scale ranged from 2.5–2.9 among adults, with a mean summary score of 13.3 (SD 5.0). Among adolescents, individual item mean scores ranged from 2.5–2.8, with a mean summary score of 13.1 (SD 5.0). There were no significant differences in summary score between adults and adolescents. Table 3 reports mean summary scores for adolescents and adults, by race & ethnicity and by gender identity. Black, non-Hispanic adults had significantly higher summary scores compared to white and other race respondents. Also, there was a trend toward significance for higher scores among Black, non-Hispanic adolescents compared to those who were white or indicated other race. There were no significant differences in mean summary score by gender identity.

## Discussion

We shortened and modified the GBMMS to measure mistrust of medical research and medical researchers, specifically. The six items we modified were from all three of the original GBMMS subscales (suspicion, disparities, and lack of support). Our results indicate both adults and adolescents may have had difficulty with the reverse-scored item. Eliminating this item resulted in improved reliability for both adults and adolescents, indicating that the five-item modified scale is reliable and valid for measuring research mistrust with American adolescents and adults of adolescents of diverse racial and ethnic identities. In support of predictive validity, scores on the modified GBMMS scale were significantly higher among adults who were Black and trended in this direction among adolescents who were Black compared to those from other races/ethnicities.

We tested this modified scale in preparation for an HIV trial, during which it was used successfully [20]. Medical mistrust, reluctance to participate in clinical research, and underrepresentation of persons of color in research have also been studied and documented in a wide range of health research contexts beyond HIV and sexually transmitted infections (e.g. Alzheimer’s disease [25], biobanking of human tissue [26], and cardiovascular research [27]). It will be important to evaluate the predictive validity of the modified GBMMS scale, therefore, in the context of other health-related research. For example, mistrust of biomedical research has been a significant barrier to important public health interventions, most notably vaccines. Vaccine hesitancy is, in part, related to the research undertaken to demonstrate efficacy and safety [28], and has resulted in excess morbidity and mortality [29]. The SARS-CoV-2 pandemic brings renewed attention to mistrust among racial and ethnic minorities [30] and among anti-vaccination groups, with critical implications for pandemic control [29]. Johnson, Velásquez, Restrepo et al. [29] used social media data to examine the role of mistrust-fueled anti-vaccination sentiments and the system-level reach between anti-vaccination groups and clusters of individuals who are undecided about vaccination. They found that compared to pro-vaccination groups, anti-vaccination groups are more central to the network and engage more frequently with undecided clusters, they have a larger number of sites which increase opportunities for engagement with undecided persons, and their messages are diversified (e.g. safety concerns, conspiracy theories about the origins of SARS-CoV-2, as well as rejection of pharmaceuticals) and therefore may appeal to multiple sectors of the population. These results underline the importance of documenting and addressing mistrust in order to meet public health goals, generally, and resolve health disparities, specifically.

However, the documentation of mistrust is simply the first step toward addressing its relationship to low research participation and subsequent racial disparities in health outcomes. Researchers must work to build trust with communities that have been historically mistreated in health care and health research contexts [1]. At the interpersonal level, research participants indicate the following strategies could build trust: using members of the community to recruit and enroll participants, having recruiters match the race/ethnicity of the target population, continuity in engagement with communities, and transparency [31]. At the institutional level, they report strict oversight of a study is essential for building trust. They suggest an oversight committee would ensure the study had potential to improve health for the benefit of all patients, sanction researchers who violated ethical principles, and maintain data security to the extent possible [26].

There are several limitations to this study. First, we used a non-random sample of volunteers who are panelists for a large survey research company. These volunteers cannot be assumed to be representative of potential health research participants, particularly because they are clearly willing to participate in social research surveys. Second, parents invited their adolescents to complete the surveys, and we have no way to verify that the adolescent did in fact complete the survey rather than the parent. This is a limitation of any online survey research, and not unique to our study. Finally, this is the first test of the psychometric properties of the modified GBMMS to specifically measure research mistrust. Further in vivo use will provide useful comparisons to this study.

## Conclusion

A shortened and modified version of the GBMMS to measure research mistrust demonstrates reliability and validity for use with diverse adolescents and parents of adolescents. Moving forward, this tool could be used to measure research mistrust among populations experiencing health related disparities and low engagement in health research. Importantly, it could also be used to assess research mistrust at the beginning of studies and at their conclusion, to determine if the suggested methods for building trust in research participants are effective.
